# Randomized prenatal and postnatal nutrient supplementation shows no long-term impact on cortical gray matter in Ghanaian children

**DOI:** 10.3389/fnhum.2025.1672317

**Published:** 2026-01-23

**Authors:** Fatimah B. Ayete Labi, Ludmila Midrigan-Ciochina, Elizabeth L. Prado, Seth Adu-Afarwuah, Kathryn G. Dewey, Charles D. Arnold, Adom Manu, Seth Kwadjo Angmorterh, John Arko-Mensah, Mavis Osipi Mensah, Helena Nti, Lois M. Donkor Aryee, Yaw Boateng Mensah, Becky Amponsaa Appiah, David Atawone, Norbert Azantilow, Brietta M. Oaks, Benjamin Amponsah, Paul D. Hastings, Amanda E. Guyer

**Affiliations:** 1 Department of Biological, Environmental and Occupational Health, School of Public Health, College of Health Sciences, University of Ghana, Accra, Ghana; 2 Department of Medical Imaging, University of Health and Allied Sciences, Ho, Ghana; 3 Center for Mind and Brain, University of California, Davis, Davis, CA, United States; 4 Institute for Global Nutrition, Department of Nutrition, University of California, Davis, Davis, CA, United States; 5 Department of Nutrition and Food Science, University of Ghana, Accra, Ghana; 6 Department of Population, Family and Reproductive Health, School of Public Health, University of Ghana, Accra, Ghana; 7 Department of Nutrition and Dietetics, University of Health and Allied Sciences, Ho, Ghana; 8 Korle-bu Teaching Hospital, Accra, Ghana; 9 Department of Radiology, International Maritime Hospital, Tema, Ghana; 10 Department of Nutrition, University of Rhode Island, Kingston, RI, United States; 11 Department of Psychology, University of Ghana, Accra, Ghana; 12 Department of Psychology, University of California, Davis, Davis, CA, United States; 13 Department of Human Ecology, University of California, Davis, Davis, CA, United States

**Keywords:** cortical, gray matter thickness, gray matter volume, maternal and child nutrition, nutrient supplementation, SQ-LNS, subcortical

## Abstract

**Introduction:**

Maternal and child undernutrition are linked to atypical brain development in children. Provision of pre- and post-natal small-quantity lipid-based nutrient supplements (SQ-LNS) has been shown to positively impact children’s growth and development. It is unknown, however, whether SQ-LNS affects child brain morphology.

**Methods:**

The present study used data from the International Lipid-based Nutrient Supplements randomized controlled trial in Ghana. Participants were 231 children (*M_*age*_* = 10.6 years; 49.4% female) exposed to maternal iron and folic acid supplements prenatally (*n* = 113, control group) or maternal SQ-LNS prenatally until 6 mo postpartum and child SQ-LNS from age 6 to 18 months (*n* = 118, SQ-LNS group). Children underwent magnetic resonance imaging (MRI) of brain anatomy. Primary outcomes were total gray matter volume, cortical gray matter thickness, and cortical gray matter volume assessed with whole-brain analyses. Secondary outcomes were thickness and volume of *a priori* specified cortical and subcortical regions assessed with region-of-interest (ROI) analyses. Basic and full covariate models were tested and corrected for multiple comparisons.

**Results:**

Whole-brain analyses revealed no significant differences between groups in total gray matter volume or cortical gray matter thickness or volume. Cortical ROI analyses showed the SQ-LNS versus control group had greater right caudal anterior cingulate cortex (ACC) thickness [mean (95%CI): 2.78 (2.73, 2.84) vs. 2.72 (2.67, 2.77); effect size = 0.21] and reduced left rostral ACC volume [2575.1 (2477.3, 2672.8) vs. 2678.74 (2568.7, 2788.8); effect size = 0.18]. Subcortical ROI analyses showed the SQ-LNS versus control group had greater volume of the left pallidus [1794.45 (1759.10, 1829.80) vs. 1726.13 (1685.05, 1767.21); effect size = 0.33] and right nucleus accumbens [751.54 (729.83, 773.24) vs. 705.73 (684.21, 727.24); effect size = 0.39]. Significant group differences did not hold after correction for multiple comparisons.

**Discussion:**

In this cohort, pre- and post-natal SQ-LNS supplementation did not significantly alter total or cortical gray matter thickness and volume at 10 years, though secondary ROI analyses indicated subtle, non-robust differences in selected regions.

## 1 Introduction

Adequate nutrition from conception through childhood is critical for brain development given the tremendous growth that occurs over those periods (Black et al., 2013; Guyer and Nordahl, 2025). The first 1,000 days after conception is a particularly vulnerable period for undernutrition because of the lasting negative consequences it can have on children’s development (Black et al., 2013). Inadequate maternal and child nutrition have been linked to atypical brain development in children (Zou et al., 2021) with negative consequences for children’s growth, academic performance, cognitive and socio-emotional development, morbidity and mortality risk, and productivity in adulthood (Boah et al., 2019; Prado and Dewey, 2014; Zou et al., 2021). Poor child outcomes due to inadequate nutrition in early life are of public health concern particularly in low- and middle-income countries (LMICs) such as Ghana where diets mainly comprise grains, cereals and tubers (Arimond et al., 2015), increasing likelihood of essential fatty acid (EFA) deficiency.

One approach to reduce children’s risk for inadequate nutrition is the provision of small-quantity lipid-based nutrient supplements (SQ-LNS) to pregnant women and their infants during the first 1,000 days of life (Arimond et al., 2015; Dewey et al., 2021; Prado and Dewey, 2014). SQ-LNS were formulated to provide the recommended dietary allowance of multiple micronutrients including iron and folic acid, plus EFAs, all of which are critical for early neurodevelopment (Prado and Dewey, 2014). Folic acid is essential for neural tube formation, cellular proliferation, and facilitating the brain’s structural integrity, which have downstream effects on cognitive and verbal skills (Chen et al., 2023). Iron aids in the early development of neural systems that mediate affect, learning, memory capacity, and information processing (Georgieff, 2023). Fatty acids (FAs) are vital for prenatal gray matter development by influencing neurogenesis, synaptogenesis, and cell function (Huffman et al., 2011; Ogundipe et al., 2018; Zou et al., 2021). With rapid accumulation of FAs in the last trimester and first 2 years of life (Lauritzen et al., 2016), FAs deficiency can cause lasting changes in brain development (Ogundipe et al., 2018). While SQ-LNS includes iron and folic acid, it has a high lipid content because an early trial in Ghana indicated that the EFA omega 3 alpha-linolenic acid (ALA) was promotive of infant linear growth in those given SQ-LNS versus micronutrients only (Adu-Afarwuah et al., 2007). It is unknown, however, whether provision of maternal and infant SQ-LNS affects brain morphology in late childhood.

The iLiNS-DYAD-Ghana randomized controlled trial (RCT) aims to understand how SQ-LNS impacts child outcomes across different developmental periods and a range of domains. SQ-LNS versus multiple micronutrient supplementation has been shown to impact children’s general physical growth and development (Dewey et al., 2021; Prado et al., 2021), including among children in the current cohort (Adu-Afarwuah et al., 2016; Bentil et al., 2024; Nti et al., 2024). Prenatal and postnatal SQ-LNS provision was linked to linear growth at age 9–11 years among female children and children whose mothers were not overweight (Bentil et al., 2024). SQ-LNS provision also led to on-time pubertal maturation age 11–13 years among females and for children raised in less resourced homes (Nti et al., 2024). Given that early nutrition supplementation manifests in these indices of physical growth, SQ-LNS effects may also extend to brain growth in late childhood, particularly given evidence of continued growth in cortical thickness, and cortical and subcortical volumes at this age (Lange, 2012; Lenroot and Giedd, 2008; Shaw et al., 2008).

Work using animal models has linked nutrient supplementation to neurogenesis and synaptogenesis, increased levels of long-chain polyunsaturated fatty acids (LC-PUFAs) in brain tissue, and larger cortical and subcortical gray matter volume (Cutuli et al., 2014; Cutuli et al., 2016; Su et al., 1999). In humans, most neuroimaging studies of the effects of nutrient supplementation on brain structure have been conducted with adult and elderly samples (McNamara et al., 2018), with a handful of correlational or RCT studies involving infants or children. Among children, prenatal exposure to higher concentrations of maternal omega-3 (ω-3), but not ω-6, LC-PUFAs during gestation exhibited non-linear associations with greater total gray and white matter volumes at age 10 years and was associated with higher academic scores when offspring were age 12 years (Zou et al., 2021). Thus, higher FA concentrations *in utero* may support children’s subsequent academic performance and brain development; however, without an RCT, causality of effects cannot be determined. Two RCTs have assessed effects of prenatal FA supplementation to mothers in high-income countries on their offsprings’ gray matter development. At age 1 month (adjusted for gestational period), male infants of mothers receiving LC-PUFAs had larger total brain, total gray matter and cortex volumes than male infants in the control group, whereas no group differences were noted for female infants (Ogundipe et al., 2018). Conversely, maternal prenatal fish oil supplementation did not predict any brain volume indices at 10 years in a separate study (Catena et al., 2019). Thus, there is limited and mixed evidence for the associations of early-life lipid-based nutrient supplementation on child brain outcomes.

There are important limitations of the studies conducted to date on whether early-life nutrition supplementation promotes children’s brain development. First, the sample sizes of the existing RCT studies may have been too small to provide sufficient statistical power to detect small-to-moderate effects (Button et al., 2013). Second, participants of the existing studies were from high income countries where rates of nutritional deficiencies are lower than in LMICs (Lemoine and Tounian, 2020). Third, prior studies focused on supplementation provided only in the gestational period, limiting understanding about direct supplementation to the young child. It is important to assess both prenatal and postnatal nutrient supplementation for both mothers and infants given that rapid brain development continues after pregnancy, with the first 2 years of life being a crucial period of brain development (Black et al., 2013). The current study sought to address these issues by examining in an LMIC setting the long-term impact of SQ-LNS provision to both pregnant women and their infants on the child’s later brain development.

Using the iLiNS-DYAD trial in Ghana, we assessed the impact of prenatal and postnatal SQ-LNS exposure versus a control condition on the gray matter of children a decade later, at the cusp of early adolescence. Primary outcomes were total gray matter volume, and cortical gray matter thickness and volume assessed with whole-brain analyses. Secondary outcomes were thickness and volume of *a priori* specified cortical and subcortical region of interest (ROI) analyses. We chose these ROIs because of their involvement in cognitive and social-emotional functions supported by gray matter development. We selected cortical regions that subserve cognitive control, emotion regulation, working memory and object-recognition functions (i.e., middle and orbital prefrontal cortex, anterior cingulate cortex (ACC), inferior parietal, superior temporal) and subcortical regions involved in decision making, reward seeking, and emotion regulation (i.e., nucleus accumbens, caudate, putamen, globus pallidus, amygdala, and hippocampus) (Baxter and Croxson, 2012; Chan et al., 2017; Cox and Witten, 2019; Etkin et al., 2011; Rolls, 2019; Stevens et al., 2011). We hypothesized that relative to children in the control group, children who had received SQ-LNS prenatally and as infants would have greater total gray matter volume and cortical gray matter thickness and volume. In exploratory analyses of secondary outcomes, we expected the SQ-LNS versus control group to have greater thickness and volume of cortical ROIs and volume of subcortical ROIs.

## 2 Materials and methods

### 2.1 Participants

Original trial randomization for the iLiNS-DYAD Ghana study was done at the participant level via a simple randomization scheme in blocks of nine (Adu-Afarwuah et al., 2016). Group assignment was stored in opaque envelopes and upon enrollment, the participant would select one envelope from the top nine envelopes to reveal their allocation. Data collectors remained blinded to study group assignment throughout the trial and all subsequent follow-ups. All surviving participants enrolled in the iLiNS-DYAD Ghana study (Adu-Afarwuah et al., 2015) who completed the first follow-up study (*n* = 1114) at age 4–6 years (Ocansey et al., 2019) were eligible for enrollment into the second follow-up study at age 9–11 years. This included the current 10-year longitudinal follow-up study. Of 979 children enrolled in the second follow-up study, 966 completed the age 9–11-year field laboratory assessments from December 2020 to December 2021.

Of the 966 participants, 240 were randomly selected from the experimental and control groups. The experimental group included mothers who were assigned to receive SQ-LNS during pregnancy until 6 mo postpartum, whose children also received SQ-LNS directly from 6 to 18 mo of age. The control group included mothers assigned to receive iron and folic during pregnancy and placebo (low-dose calcium) from delivery until 6 mo postpartum whose children received no supplementation from 6 to 18 mo of age. Random selection was implemented by contacting participants from a randomly sorted list of participants to maintain enumerator blinding. This was done until the target of 240 participants were screened for MRI scanning eligibility using a standard questionnaire. The list was block randomized by intervention arm to increase the chances of balance by intervention arm. Ultimately 116 were selected from the control arm and 124 from the SQ-LNS arm. MRI technologists and image analysts were blinded to group assignments. MRI exclusion criteria were neurological abnormalities and any MRI contraindications including reported: (a) metal in the body (e.g., shrapnel, implanted devices); (b) pregnancy (females); (c) refusal to participate in the scan; (d) experience of distress or fear regarding the MRI; (e) claustrophobia; or (f) significant illness/disability. All 240 participants who were screened were eligible for MRI scanning and then enrolled after caregiver consent and child assent to undergo the MRI scans. One participant did not attend the scheduled scan. An *a priori* power analysis indicated that a minimum sample size of 220 participants (110 participants in each group) was required to provide 80% power to detect an effect size > 0.38 SD at *p* < 0.05 and 240 were targeted to allow for refusals.

### 2.2 Procedure

Participants were transported in the project van together with a caregiver willing to travel on the day of the MRI assessment on a 64 km journey from study sites to the International Maritime Hospital (IMaH) in Tema, Ghana. Trained research assistants explained the MRI procedure to the participants and their caregivers. Participants were administered an MRI screening form from the ImaH imaging department to check for any MRI contraindications (e.g., metallic implants, pace marker). Participants also went through a metal detector to ascertain that they were metal free after changing into a hospital gown. Participants were then acclimated to the MRI procedures by watching videos of children undergoing MRI scans and given the opportunity to ask questions about the procedures. The videos were in three local languages (Twi, Ewe, and Dangbe) and Ghanaian accent English, which were commonly spoken in the study area.

### 2.3 Measures

#### 2.3.1 Sample characteristics

Maternal and household baseline characteristics were collected at time of enrollment into the main study. Child characteristics were also collected at birth. Child’s sex, puberty stage, handedness, and number of years in formal education, were collected at the 9–11-year-old follow-up. These four measures were included as covariates in statistical models to mitigate any associations with significant differences in brain structure, function, and developmental trajectories that could confound results. Sex and puberty stage were included because genetic and hormonal differences drive sex-specific patterns of brain maturation during adolescence (Blakemore et al., 2010; Gaillard et al., 2021). Handedness was included because left- and right-handed individuals show differences in brain structure, lateralization, and functional organization (Tejavibulya et al., 2022). Educational attainment may reflect differences in cognitive stimulation, experiences, or socioeconomic factors that influence brain outcomes (van Atteveldt et al., 2018). The primary caregiver reported the number of years of formal education completed by the child as the current grade of the child, which ranged from elementary school grades 1–5. This measure was dichotomized to “below grade 3” and “grade 3 and above.” Children’s pubertal development was measured using the Petersen Pubertal Development Scale (PDS) (Petersen et al., 1988), a 10-item (5 for boys and 5 for girls) questionnaire about children’s physical maturation completed by both the primary caregiver and the child. Participants responded to each item on the PDS using a 4-point Likert scale that described their current stage for 5 pubertal milestones each for boys and girls; responses were summed to create a total score ranging from 5 (puberty not begun) to 20 (puberty complete). The PDS was also used to record the biological sex of the child. Lastly, a modified version of the Edinburgh Handedness Inventory (Oldfield, 1971), which included 6 items adapted to the local context in Ghana, and for the appropriate age (e.g., 9–11 years old), was used to calculate a laterality index (handedness) of the child’s dominant hand use.

#### 2.3.2 Neuroimaging data, image acquisition and preprocessing

Structural MRI data were collected using a 3T Philips Ingenia system at the Radiology department of the IMaH at Tema in Ghana, using a standard 16-channel adult-size head coil. A magnetization prepared rapid gradient echo (MPRAGE) sequence was used to create high-resolution T1-weighted images of the brain (see Supplementary Methods for acquisition parameters). The T1-weighted images were screened for quality during and after image acquisition. Raw images (.dcm) were converted to NIFTI format using the “dcm2nii” function from www.nitrc.org.

**FIGURE 1:**
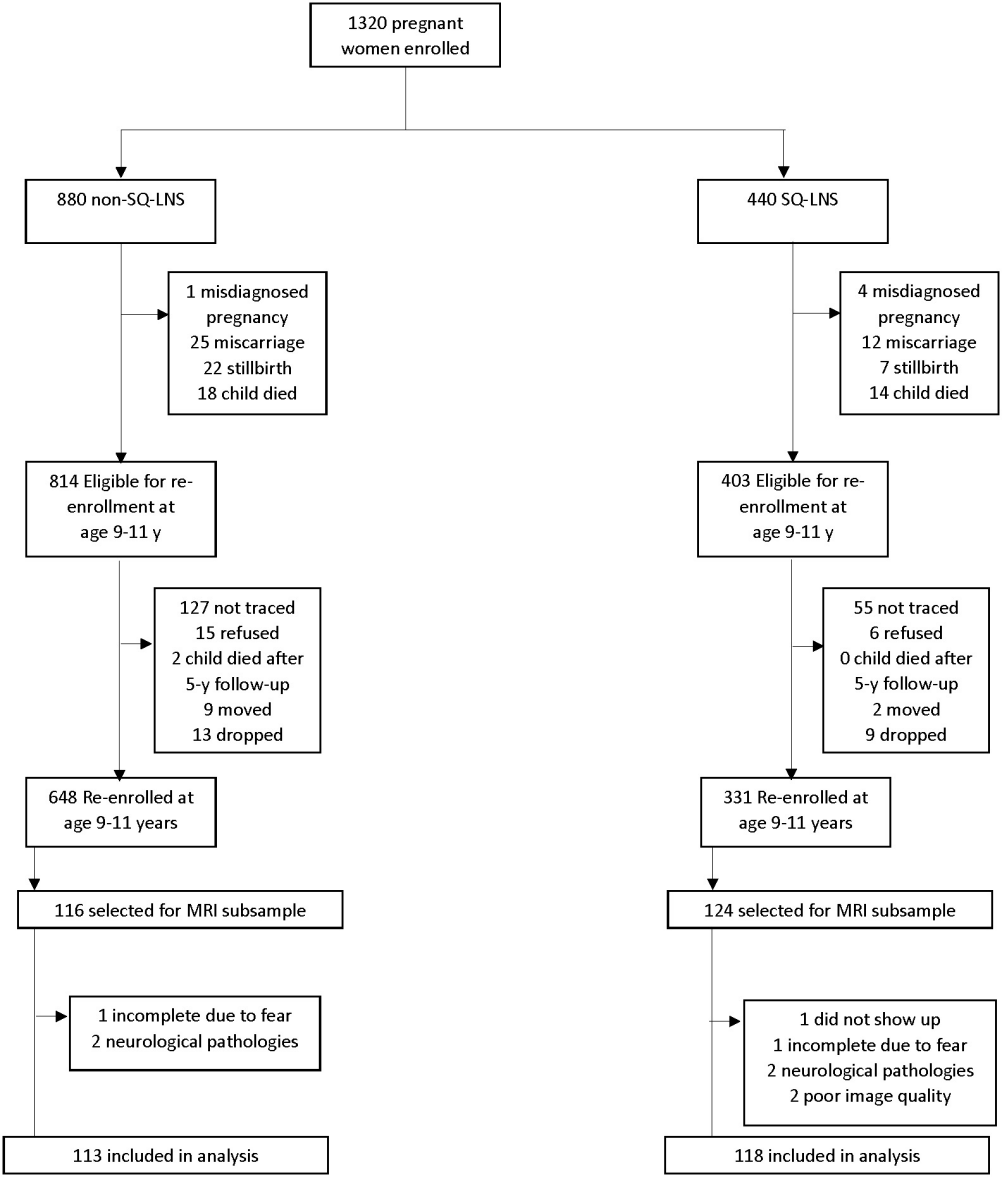
Flowchart of child’s eligibility, enrollment, and data collection for the small-quantity lipid-based nutrient supplements (SQ-LNS) randomized controlled trial in Ghana.

T1-weighted image preprocessing, reconstruction, and volumetric and cortical parcellation were carried out with the “recon-all” command from the FreeSurfer image analysis suite, version 7.2.0.^1^ After registration to the common template (freesurfer average-fsaverage), the data were smoothed using a 10 mm 2D Gaussian smoothing kernel (Fischl et al., 1999). Quality control (QC) assessments were performed visually for all 239 participants with a scan after data acquisition and preprocessing steps. The QC led to excluding data of participants with incomplete T1-weighted images from scans shortened due to child’s fear (*n* = 2), and with excessive image blurring due to motion, or significant mechanical artifacts (*n* = 2). Additionally, participants’ neuroimaging data were excluded from analysis if any neuropathology was identified in the scan by a radiologist (*n* = 4). Figure 1 shows the tracking of inclusion and exclusion criteria. No adverse events related to MRI were reported.

Total intracranial volume (TIV) values for each participant who passed QC were obtained from the FreeSurfer’s automated segmentation pipeline and standardized in Excel to be included in all whole-brain and ROI analyses. Total gray matter volume values were obtained from the FreeSurfer output, which provided an overall measure of gray matter volume including all cortical and subcortical regions. These values were standardized before inclusion in analyses. Cortical gray matter thickness and volume values for ROIs including left and right caudal and rostral middle frontal cortex, medial and lateral orbitofrontal cortex, caudal and rostral ACC, inferior parietal cortex, and superior temporal cortex were obtained from FreeSurfer output (*N* = 16 ROIs). Subcortical volume values for ROIs, including left and right amygdala, hippocampus, thalamus, caudate, pallidum, putamen, and nucleus accumbens were also obtained from the FreeSurfer output (*N* = 14 ROIs).

Primary outcomes in this study include total gray matter volume, cortical thickness, and cortical volume. Secondary outcomes include selected ROIs of cortical thickness and volume and ROIs of subcortical volumes.

### 2.4 Statistical analyses

The statistical analysis plan for this study was preregistered at https://osf.io/bmv9d/.

#### 2.4.1 Baseline characteristics and covariates

Two sample *t*-tests were used to compare the SQ-LNS and control groups on maternal, child, and household baseline characteristics, as well as sample characteristics selected as covariates due to their putative associations with brain morphology development.

#### 2.4.2 Brain morphology

All whole-brain and ROI-based statistical models predicting brain gray matter indices were initially fitted with standardized total intracranial volume (mm^3^) as a nuisance covariate (basic model). Second similar models were then fitted with child biological sex (male/female), child’s formal (years) education, age 9–10 pubertal development score, and laterality index (handedness) in addition to standardized total intracranial volume (mm^3^) to control for them as nuisance covariates (full model). Similarly, basic and full regression models were conducted to test for SQ-LNS and control group differences in total gray matter volume. Our preregistered whole-brain analyses were conducted using FreeSurfer, and all the *ad hoc* regression analyses were conducted using Stata version 15.0.

##### 2.4.2.1 Cortical thickness and volume

Whole-brain vertex-wise general linear models (basic and full) were conducted to test differences between the SQ-LNS and control groups in primary outcomes of cortical gray matter thickness and volumes. Following standard practice, a cluster-wise correction threshold of 3.0 at *p* < 0.05 was applied to whole-brain analyses (Greve and Fischl, 2018). Cluster-wise correction thresholding is essential to correct for the multiple statistical comparison that occur at the voxel level in neuroimaging analysis.

ROI regression analyses (basic and full) were conducted to test for differences between the SQ-LNS and control group in the thickness and volume of selected cortical regions, including left and right caudal and rostral middle frontal cortex, medial and lateral orbitofrontal cortex, caudal and rostral ACC, inferior parietal cortex, and superior temporal cortex (secondary outcomes). Multiple comparison correction was performed using the false discovery rate (FDR) correction at *p* < 0.05 for the 16 selected cortical ROIs.

##### 2.4.2.2 Subcortical volume

ROI regression analyses (basic and full) were conducted to test for differences between the SQ-LNS and control group in the volumes of the left and right amygdala, hippocampus, thalamus, caudate, pallidus, putamen, and nucleus accumbens (secondary outcomes). Multiple comparison correction was performed using the FDR correction at *p* < 0.05 for the 14 selected subcortical ROIs.

## 3 Results

### 3.1 Baseline characteristics

The flow of participation in the current study is represented in Figure 1.

Table 1 shows the maternal, child, and household characteristics of mothers and children selected for MRI assessment. Mothers in the SQ-LNS group had higher pre-pregnancy body mass index than the control group (*p* = 0.03). Additionally, children in the SQ-LNS group had higher birth weight as compared to the control group (*p* = 0.03). There were no other significant differences between the SQ-LNS and control groups on other maternal or household measures. Supplementary Table 1 indicates no significant differences in baseline characteristics of participants in the current study and the main study.

**TABLE 1:** Maternal, child, and household baseline characteristics.

Characteristics	Total sample	SQ-LNS *N* = 124	Control *N* = 116	*t*-statistic	*p*-value
**Maternal**					
Age at enrollment, y, mean (SD)	26.7(5.5)	26.9(5.0)	26.5(5.0)	0.6	0.6
Educational level, n (%)					0.1
Below high school	207(86.3)	103(83.1)	104(89.7)		
High school and above	33(13.8)	21(16.9)	12(10.3)		
Marital status n (%)					0.6
With partner	216(90.0)	113(91.1)	103(88.8)		
Without partner	24(10.0)	11(8.9)	13(11.2)		
Pre-pregnancy BMI, mean (SD)	24.4(4.3)	24.9(4.6)	23.7(4.0)	2.2	**0.03**
Primiparous, n (%)					0.8
No previous child	83(34.5)	42(33.9)	41(35.3)		
Previous child	157(65.4)	82(66.1)	75(64.7)		
Hemoglobin level, mean (SD)	111.1(12.0)	110.8(12.3)	111.3(11.8)	−0.3	0.8
**Child**					
Child weight at birth, kg, mean (SD)	3.00(0.41)	3.06(0.42)	2.94(0.39)	2.1	**0.03**
**Household**					
Household asset index, mean (SD)	0.05(0.93)	0.07(0.88)	0.04(0.98)	0.21	0.8
Household food insecurity access score, mean (SD)	2.1(4.0)	1.8(3.5)	2.5(4.4)	1.4	0.2
Household water source, n (%)					1.0
Improved	238(99.2)	123(99.2)	115(99.1)		
Unimproved	2(0.8)	1(0.8)	1(0.9)		
Household toilet facility, n (%)					0.5
Improved	229(95.8)	119(96.8)	110(94.8)		
Unimproved	10(4.2)	4(3.3)	6(5.2)		

SQ-LNS, Small quantity lipid-based nutrient supplementation. Significant *p*-values are in bold.

### 3.2 Covariates

There were no significant group differences in child’s total standardized intracranial volume, handedness, pubertal stage, or biological sex (Table 2). More children in the SQ-LNS than the control group had reached a formal education level of at least grade 3 by age 10 years.

**TABLE 2:** Means, standard deviations, and group differences in covariates.

Variable	SQ-LNS *N* = 118	Control N = 113	t-statistic	95% CI	*p*-values
Total intracranial volume (mm^3^)	1,275,395	1,253,233	−1.4	1,248,971–1,280,137	0.2
Handedness	90.0	91.4	0.3	85.6–95.8	0.8
Pubertal score	7.4	7.5	−0.6	7.31–7.6	0.6
Biological sex (male)	51.7(61/118)	50(56/112)			
Current school grade (Below Grade 3)	18.6(22/118)	22.3(25/112)			

SQ-LNS, Small quantity lipid-based nutrient supplementation.

### 3.3 Brain morphology

Among the brain morphology primary and secondary outcomes, several strong correlations were found among cortical thickness indices, cortical volumes, and subcortical volumes (see Supplementary Tables 2–4, respectively).

#### 3.3.1 Total gray matter volume

Children in the SQ-LNS and control groups did not differ significantly on total gray matter volume in either the basic (*p* = 0.6) or full (*p* = 0.5) model (Table 3).

**TABLE 3:** Group differences in total gray matter volume.

Variable	SQ-LNS *N* = 118		Control *N* = 113		Basic model	Full model
	Mean	95% CI	Mean	95% CI	*p*-value	*p*-value
Total gray matter volume, *mm*^3^	637181.8	626566.7–647796.9	630989.8	620755.4–641224.2	0.6	0.5

SQ-LNS, Small quantity lipid-based nutrient supplementation; basic model included: Controlling for standardized total intracranial volume; full model included; Controlling for child biological sex (male/female), child’s formal (years) education, pubertal development score, and laterality index (handedness) and standardized total intracranial volume (mm^3^).

#### 3.3.2 Cortical gray matter thickness and volume

##### 3.3.2.1 Whole-brain analyses

**FIGURE 2:**
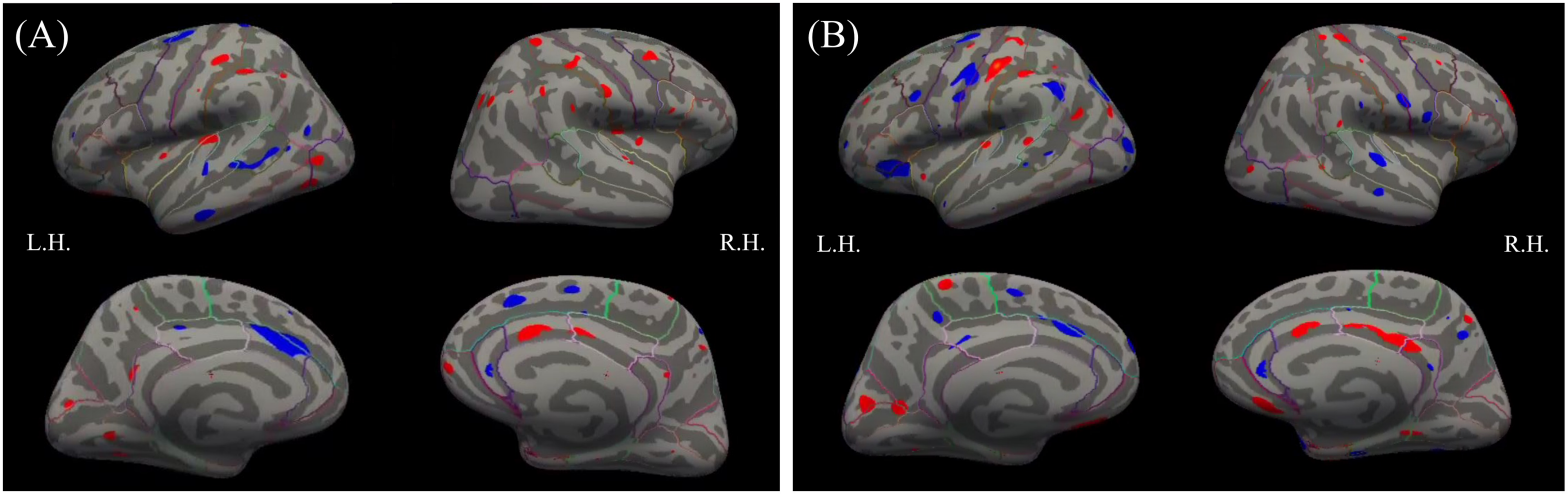
Results from the **(A)** basic (left panel) and **(B)** full (right panel) models for the whole-brain analyses contrasting the SQ-LNS group to the control group on cortical thickness. Multiple positive (red) and negative (blue) effects were found at *p* < 0.01 (uncorrected). Left (LH) and right (RH) hemispheres are shown. SQ-LNS, small-quantity lipid-based nutrient supplements.

The basic model showed multiple positive (red) and negative (blue) effects of SQ-LNS on cortical thickness in both hemispheres. The SQ-LNS group had thicker cortical gray matter than the control group in multiple regions (red), including the left insula, right caudal middle frontal gyrus, and right caudal anterior cingulate cortex (Figure 2A). After correcting for multiple comparisons at a cluster-wise threshold of 3.0 and cluster-wise *p* < 0.05, none of these group differences remained significant. Similarly, no group differences were significant when all covariates were included in the full models (Figure 2B).

**FIGURE 3:**
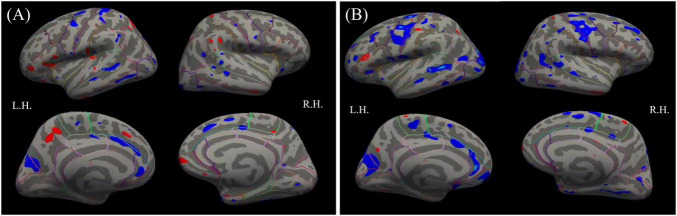
Results from the **(A)** basic (left panel) and **(B)** full (right panel) models for the whole-brain analyses contrasting the SQ-LNS group to the control group on cortical volume. Multiple positive (red) and negative (blue) effects were found at *p* < 0.01 (uncorrected). Left (LH) and right (RH) hemispheres are shown. SQ-LNS, small-quantity lipid-based nutrient supplements.

The basic model showed multiple negative (blue), and a few positive (red), effects of SQ-LNS on cortical volume in both hemispheres. The SQ-LNS group had smaller volume of cortical gray matter than the control group in multiple regions (blue), including the left and right precuneus, and left rostral ACC (Figure 3A). After correcting for multiple comparisons at a cluster-wise threshold of 3.0 and cluster-wise *p* < 0.05, none of these group differences remained significant. Similarly, no group differences were significant when all covariates were included in the full models (Figure 3B).

##### 3.3.2.2 Region-of-interest analyses

Regression analyses were performed on the thickness of specific cortical gray matter ROIs (Table 4). The basic model showed that the SQ-LNS group had greater thickness of the right caudal ACC region (2.8, 2.7–2.8) than the control group (2.7, 2.7–2.8) at a trend level of significance (*p* = 0.05), which remained in the full model (*p* = 0.05) (see Supplementary Figure 1). However, this group difference was small and no longer significant when the multiple comparisons correction was applied. No group differences in the thickness of any other cortical gray matter ROI were found to be significant.

**TABLE 4:** Group differences in gray matter thickness of cortical regions of interest.

Cortical gray matter thickness	SQ-LNS		Control		Basic model		Full model	
	Mean	95%CI	Mean	95%CI	*p*-value (uncorrected)	*p*-value (corrected)	*p*-value (uncorrected)	*p*-value (corrected)
Lt. Caudal middle frontal	2.6	2.5–2.6	2.6	2.5–2.6	0.7	0.9	0.6	0.8
Rt. Caudal middle frontal	2.5	2.5–2.6	2.5	2.5–2.5	0.4	0.9	0.4	0.8
Lt. Rostral middle frontal	2.5	2.5–2.5	2.5	2.5–2.5	0.7	0.9	0.7	0.8
Rt. Rostral middle frontal	2.4	2.4–2.4	2.4	2.4–2.4	1.0	1.0	1.0	1.0
Lt. Lateral orbitofrontal	3.1	3.1–3.1	3.1	3.1–3.1	0.6	0.9	0.6	0.8
Rt. Lateral orbitofrontal	2.9	2.9–2.9	2.9	2.9–3.0	0.4	0.9	0.4	0.8
Lt. Medial orbitofrontal	2.8	2.8–2.8	2.8	2.7–2.8	0.4	0.9	0.3	0.8
Rt. Medial orbitofrontal	2.8	2.8–2.8	2.8	2.8–2.8	0.4	0.9	0.4	0.8
Lt. Caudal anterior cingulate	2.8	2.8–2.9	2.9	2.9–2.9	0.1	0.5	0.1	0.5
Rt. Caudal anterior cingulate	2.8	2.7–2.8	2.7	2.7–2.8	**0.05**	0.5	**0.05**	0.5
Lt. Rostral anterior cingulate	3.2	3.1–3.2	3.2	3.1–3.2	1.0	1.0	1.0	1.0
Rt. Rostral anterior cingulate	3.2	3.2–3.3	3.2	3.2–3.3	0.5	0.9	0.5	0.8
Lt. Inferior parietal	2.3	2.3–2.4	2.4	2.3–2.4	0.7	0.9	0.7	0.8
Rt. Inferior parietal	2.5	2.5–2.5	2.5	2.5–2.5	0.7	0.9	0.7	0.8
Lt. Superior temporal	3.1	3.0–3.1	3.1	3.1–3.1	0.5	0.9	0.5	0.8
Rt. Superior temporal	3.1	3.1–3.2	3.2	3.1–3.2	0.4	0.9	0.4	0.8

SQ-LNS, Small quantity lipid-based nutrient supplementation; basic model included: Controlling for standardized total intracranial volume; full model included; Controlling for child biological sex (male/female), child’s formal (years) education, pubertal development score, and laterality index (handedness) and standardized total intracranial volume (mm^3^); CI, Confidence interval; Lt, Left; Rt, Right. Significant *p*-values are in bold.

Regression analyses were performed on the gray matter volume of specific cortical ROIs (Table 5). The basic model showed that the SQ-LNS group had significantly smaller volume (2575.1, 2672.8–19240) of the left rostral ACC region than the control group (2678.7. 2568.7–2788.8) (*p* = 0.03), which remained significant in the full model (*p* = 0.01) (see Supplementary Figure 2). However, this group difference was small and no longer significant when multiple comparisons correction was applied. No group differences in the volumes of any other cortical gray matter ROIs were found to be significant.

**TABLE 5:** Group differences in gray matter volumes of cortical regions of interest.

Cortical gray matter volume	SQ-LNS		Control		Basic model		Full model	
	Mean	95%CI	Mean	95%CI	*p*-value (uncorrected)	*p*-value (corrected)	*p*-value (uncorrected)	*p*-value (corrected)
Lt. Caudal middle frontal	5649.5	5485.1–5814.0	5730.8	5529.2–5932.4	0.2	0.8	0.1	0.5
Rt. Caudal middle frontal	5209.5	5044.0–5375.0	5215.7	5034.6–5396.9	0.5	0.9	0.5	0.7
Lt. Rostral middle frontal	15367.8	14873.8–15861.7	15063.9	14615.4–15512.3	0.9	0.9	1.0	1.0
Rt. Rostral middle frontal	15514.7	14943.6–16085.9	15374.5	14858.5–15890.4	0.6	0.9	0.4	0.7
Lt. Lateral orbitofrontal	8316.0	8119.9–8512.2	8164.5	7996.8–8332.2	0.7	0.9	0.9	1.0
Rt. Lateral orbitofrontal	7810.9	7611.9–8009.8	7821.6	7634.0–8009.2	0.3	0.8	0.2	0.5
Lt. Medial orbitofrontal	5856.4	5713.7- 5999.0	5788.9	5652.3–5925.5	0.8	0.9	0.8	1.0
Rt. Medial orbitofrontal	6206.0	6068.5–6343.5	6066.7	5944.3–6189.1	0.4	0.9	0.4	0.7
Lt. Caudal anterior cingulate	1800.0	1705.8–1894.3	1865.4	1778.3–1952.5	0.2	0.8	1.0	0.5
Rt. Caudal anterior cingulate	2108.1	2008.8–2207.5	2065.5	1979.0–2152.0	0.9	0.9	0.9	1.0
Lt. Rostral anterior cingulate	2575.1	2477.3–2672.8	2678.7	2568.7–2788.8	**0.03**	0.4	**0.01**	0.2
Rt. Rostral anterior cingulate	2020.8	1924.0–2117.5	2016.9	1920.1–2113.6	0.4	0.9	0.4	0.7
Lt. Inferior parietal	11390.7	11064.34–11717.0	11263.4	10936.8–11590.1	0.9	0.9	0.8	1.0
Rt. Inferior parietal	14898.9	14444.2–15353.5	14841.6	14425.6–15257.7	0.4	0.9	0.4	0.7
Lt. Superior temporal	14095.5	13539.4–14198.7	13869.0	13539.4–14198.7	0.9	0.9	1.0	1.0
Rt. Superior temporal	13013.3	12703.7–13322.8	13066.4	12764.05–13368.71	0.2	0.8	0.1	0.5

SQ-LNS, Small quantity lipid-based nutrient supplementation; basic model included: Controlling for standardized total intracranial volume; full model included; Controlling for child biological sex (male/female), child’s formal (years) education, pubertal development score, and laterality index (handedness) and standardized total intracranial volume (mm^3^); CI, Confidence interval; Lt, Left; Rt = Right. Significant *p*-values are in bold.

#### 3.3.3 Subcortical gray matter volume

##### 3.3.3.1 Region-of-interest analyses

Regression analyses were conducted on the gray matter volume of specific subcortical ROIs (Table 6). The basic model showed that the SQ-LNS group had significantly larger volume of the left pallidus (*p* = 0.04) and the right nucleus accumbens area (*p* = 0.01) than the control group (see Supplementary Figures 3, 4). Only the group difference in the volume of the right nucleus accumbens was significant in the full model (*p* = 0.02). However, these group differences were small and were not robust after multiple comparisons. No group differences in the volumes of any other subcortical gray matter ROIs were found to be significant.

**TABLE 6:** Group differences in gray matter volumes of subcortical regions of interest.

Subcortical volume	SQ-LNS		Control		Basic model		Full model	
	Mean	95%CI	Mean	95%CI	*p*-value (uncorrected)	*p*-value (corrected)	*p*-value (uncorrected)	*p*-value (corrected)
Lt. Amygdala	1.713.5	1674.7 –1752.3	1689.2	1651.97 –1726.39	0.9	0.9	1.0	1.0
Rt. Amygdala	1793.8	1751.1–1836.4	1781.3	1743.98–1818.60	0.6	0.7	0.7	0.8
Lt. Hippocampus	3869.0	3795.1–3942.9	3788.7	3713.50 –3863.86	0.4	0.6	0.5	0.8
Rt. Hippocampus	3921.0	3854.4–3987.7	3852.2	3785.73 –3918.70	0.5	0.6	0.6	0.8
Lt. Thalamus	6969.7	6862.1–7077.2	6856.6	6725.38–6987.83	0.7	0.7	0.8	0.9
Rt. Thalamus	6849.7	6744.5–6954.8	6690.4	6565.65–6815.19	0.2	0.4	0.2	0.5
Lt. Caudate	3726.4	3639.9–3812.9	3582.8	3491.54–3674.06	0.1	0.4	0.1	0.4
Rt. Caudate	3958.2	3866.1–4050.3	3831.1	3739.19–3923.07	0.2	0.4	0.3	0.5
Lt. Pallidus	1794.5	1759.1–1829.8	1726.1	1685.05–1767.21	**0.04**	0.3	0.1	0.4
Rt. Pallidus	1726.9	1694.2–1759.7	1672.4	1634.24– 1710.61	0.1	0.4	0.2	0.5
Lt. Putamen	5263.1	5167.2–5359.0	5134.8	5026.89–5242.70	0.2	0.4	0.3	0.5
Rt. Putamen	5311.9	5219.8—5404.0	5172.8	5066.38–5279.19	0.2	0.4	0.2	0.5
Lt. Nucleus Accumbens	738.0	715.7–760.35	713.3	691.65–734.89	0.3	0.4	0.3	0.5
Rt. Nucleus Accumbens	751.5	729.8–773.24	705.7	684.21–727.24	**0.01**	0.1	**0.02**	0.2

SQ-LNS, Small quantity lipid-based nutrient supplementation; basic model included: Controlling for standardized total intracranial volume; full model included; Controlling for child biological sex (male/female), child’s formal (years) education, pubertal development score, and laterality index (handedness) and standardized total intracranial volume (mm^3^); CI, Confidence interval; Lt, Left; Rt, Right. Significant *p*-values are in bold.

## 4 Discussion

The present study investigated the effects of providing SQ-LNS in early life on the brain morphology of children enrolled in the iLiNS-DYAD-Ghana RCT a decade later. Children whose mothers received SQ-LNS from early pregnancy to 6 months postpartum, and then themselves received SQ-LNS from age 6 to 18 months, differed from the control group on primary outcomes of cortical thickness and volume in multiple regions based on uncorrected whole-brain analyses. However, when correction for multiple comparisons was applied, these group differences were no longer significant. Children in the SQ-LNS also did not differ from the control group in total gray matter volume. Exploratory analyses of secondary cortical ROI outcomes showed that the SQ-LNS group as compared to the control group had marginally greater thickness of the right caudal ACC, but also smaller volume of the left rostral ACC. Analyses of secondary subcortical ROIs outcomes showed that the SQ-LNS group demonstrated greater volume of all subcortical structures of interest, with significant differences in the left pallidus and right nucleus accumbens. The group differences from the ROI analyses also emerged only for the exploratory uncorrected analyses. Nonetheless, these results are still informative, given a small empirical literature from which to guide a narrower selection of ROIs and the conservative nature of the multiple-hypothesis corrections, which assume testing independence, applied to correlated outcomes. Although there were no significant differences after FDR correction, several of the regions of the brain were highly correlated. Consequently, multiple hypothesis corrections that assume independence of outcomes may be overly conservative (Streiner, 2015) resulting in inflated FDR or true effects being missed (Santosa et al., 2017; Winkler et al., 2025). Future research should consider larger harmonized RCT-neuroimaging datasets to help address this (Winkler et al., 2025). In uncorrected comparisons, we did find differences in thickness of the right caudal ACC and volumes of the left rostral ACC and pallidus and right nucleus accumbens that warrant further investigation in future research.

Whole-brain analyses, when uncorrected, showed that early-life SQ-LNS had a small effect, whereby the SQ-LNS group had thicker cortical gray matter than the control group in multiple regions, such as the left insula, right caudal middle frontal, and right caudal ACC. These regions have been implicated in interoception, motor control, executive function, cognitive processing and emotional regulation (Stevens et al., 2011; Uddin et al., 2017). It is possible that SQ-LNS contributes to these functions via gray matter development, however that would need to be empirically tested. Contrary to our predictions, however, SQ-LNS was not significantly associated with cortical thickness or volume across the whole brain after applying the cluster-wise correction to adjust for multiple comparisons. Given these null findings, it is possible that because all mothers received iron and folic acid supplementation, the roles these nutrients play in early brain development obscured the contributions of the EFAs in the lipid-based supplements received only by the SQ-LNS group. It is also possible that gray matter differences would have been more pronounced/detectable earlier in development, but in the nearly decade-long period between completing SQ-LNS supplementation and performing scans, intervening factors may have contributed to brain growth in the control group. Plausible candidates include childhood diet and stimulation through home and school environments. Future studies will be needed to examine the role of such factors in brain development following SQ-LNS supplementation.

The present findings are similar to those from two earlier studies (Catena et al., 2019; Ogundipe et al., 2018). In the RCT conducted by Ogundipe et al. (2018), no significant differences in brain volume, measured 1 month after birth, were found between infants of mothers who received brain specific fatty acid (BSFA) supplements and a control group. However, this was qualified by a sex difference whereby greater total brain, cortex, corpus callosum, and total gray matter volumes, as well as greater birth length and head circumference, were seen for infant boys in the BSFA group compared to boys in the control group. While these sex-related differences were significant at age 1 month, this may have been too early to observe all differences given rapid postnatal brain growth. It also would not have captured the postnatal supplementation effects as per our study design. Thus, the nearly decade-long difference in age at brain volume measurement between our study and Ogundipe et al.’s limits comparability of the respective results. In a different study that examined offspring at a similar age as in our study, no significant differences at age 10 years were reported in cortical or subcortical gray matter volumes between children whose mothers were randomly administered either fish oil, folic acid, a combination of fish oil and folic acid, or a placebo from gestational week 22 until delivery, paralleling our findings (Catena et al., 2019). Notably, both Catena et al.’s (2019) study and the present study found the supplementation group had larger mean total gray matter volume than the comparison group, although in neither study did this reach statistical significance. The non-significant differences could be an indication of only small effects of early-life SQ-LNS supplementation enduring to preadolescence, necessitating further follow-up to detect potentially long-lasting effects.

In the cortical ROI analysis, the uncorrected results indicated that SQ-LNS was inversely associated with volume of left rostral ACC and positively associated with thickness of right caudal ACC, although these associations were not significant after multiple comparison correction. As part of the limbic system, the ACC has long been implicated in cognitive processing and emotional regulation (Stevens et al., 2011), making it a unique area for understanding socio-emotional behaviors as adolescence begins. Due to its anatomical position and connection with other brain structures, the rostral/ventral portion is implicated in emotion processing and regulation of autonomic and endocrine response to emotions, whilst the caudal/dorsal portion is involved in cognitive processing such as reward-based decision making (Stevens et al., 2011; Vogt, 2016). Thus, our study may offer preliminary support for early-life SQ-LNS being associated specifically with increased thickness of caudal ACC and reduced volume of rostral ACC in early adolescence. This possibility should be acknowledged with caution as all significant effects were lost after multiple hypothesis correction. Nonetheless, future work should consider the ACC, and its behavioral correlates, as a brain region potentially affected by SQ-LNS. This recommendation is supported further by work documenting concurrent negative associations between whole blood omega 3 fatty acid levels and caudal ACC volume in adolescents (Darcey et al., 2019, 2020), although these associations were not robust to conservative multiple comparison correction, as in our study. To the best of our knowledge, no prior study has investigated the effect of SQ-LNS on thickness of the ACC, limiting its interpretation, but suggesting another possible feature of the ACC to target with supplementation.

Generally, subcortical structures have been linked to cognitive, affective and social functions, thereby structural alterations in subcortical regions have been associated with psychiatric and behavioral disorders such as depression and schizophrenia (Forbes and Dahl, 2012; Koshiyama et al., 2018; Paus et al., 2008). The uncorrected findings from the current study indicate that SQ-LNS is positively associated with volumes of the left pallidus and right nucleus accumbens. The nucleus accumbens is involved with the regulation of motivation, reward, and positive affect, and has been implicated in several mental health conditions such as depression, bipolar disorders, anxiety disorders, and drug abuse and addictions, among others (Xu et al., 2020). Notably, these mental health problems become more prevalent over the course of adolescence. Similarly, the pallidus communicates with several cortical and subcortical areas that subserve cognition, motor activity and reward motivation (Saga et al., 2017). Previous nutrition-based studies have only examined the association of dietary patterns with total subcortical volume (Boedhoe et al., 2021; Mou et al., 2023), such that it is not clear whether the specific small effects we observed for SQ-LNS predicting nucleus accumbens and pallidus volumes have precedent in literature. Due to the relation of nucleus accumbens and pallidus to cognitive, affective, social and motor functions, nutrition-related alterations in these structures may have implications for multiple aspects of adolescent mental health, particularly those that involve reward motivation. Further nutrition research targeting these subcortical regions may be warranted.

Importantly, this study only considered direct effects of the intervention and did not assess whether life contexts or other characteristics may have made certain children more or less responsive to the intervention. For example, findings from an earlier iLiNS-DYAD-Ghana RCT study showed greater effect of SQ-LNS on behavioral outcomes among children from less resourced homes (Ocansey et al., 2019); whether this may also be the case for brain development has yet to be determined. Likewise, at baseline, higher pre-pregnancy maternal BMI and higher child birthweight in the SQ-LNS group may have facilitated its effectiveness on various child outcomes or have genetic correlates, possibilities that future work should explore. Additionally, as study participants transition into adolescence, future studies should also assess the impact of puberty on brain morphology at a later age such as 11–13 years, as SQ-LNS has been associated with pubertal status of girls at 11–13 years of age (Nti et al., 2024). Also, future studies should investigate how sex differences influence brain development as sex has been shown in previous studies to be associated with differences in brain development (Catena et al., 2019; Darcey et al., 2020; Ogundipe et al., 2018) as well as an effect modifier in previous reports of the impact of SQ-LNS (Bentil et al., 2024; Nti et al., 2024). The group differences found for the uncorrected results suggest a more targeted set of brain regions for which to examine the influence of the home environment, pubertal development, and sex as well as their modification of SQ-LNS effects on these brain regions. Lastly, generalizability of the present findings should be interpreted in populations with similar nutrition-related features (e.g., maternal BMI).

### 4.1 Strength and limitations

The current study has several strengths and limitations. To the best of our knowledge, this is the first RCT in Africa, and amongst the few in the world, where SQ-LNS was administered prenatally through to 18 months postnatal and then brain structure was assessed 10 years later. The present study had a scan success rate of 99%, high-quality MRI data, and a compliance rate higher than reported in earlier studies (Everts et al., 2020; Sowell et al., 2004). The non-significant results for cortical thickness and volume in whole-brain analysis cannot be attributed to low compliance, or poor data quality. As one of the first MRI studies of a large sample of children in sub-Saharan Africa, this study also demonstrates the viability of MRI for developmental neuroscience research in such settings. Unlike cross-sectional studies which preclude understanding developmental trajectories, the present study employed an RCT study design that reduced bias and balanced participants’ characteristics whilst providing a robust sampling design to examine cause-effect relations. The current study was well-powered with a relatively large sample size. Finally, the current study employed a robust statistical threshold of 3.0 (*p* = 0.001) to reduce the possibility of attaining false positive results for the whole-brain analyses and an FDR correction across a large set of regions for the ROI analyses.

Despite the strengths of the study, no neuroimaging assessment was performed in early childhood when the impact of SQ-LNS on behavioral measures has been reported (Ocansey et al., 2019). This makes it impossible to ascertain whether there may have been impacts of SQ-LNS on brain structure during children’s early years, which may have become less evident later in development among children in the comparison condition. Children who remained in the cohort a decade later may also have systematic differences, although no differences in baseline characteristics were found between children in the current MRI study and those in the main study (Supplementary Table 1). Additionally, because this study is the first of its kind, there was limited literature to guide more specific selection of ROIs, such that the small effects observed in ROI analyses were not robust to correction for multiple comparisons. Nevertheless, this study may serve to inform more precisely targeted neuroimaging analyses in future studies of the effects of nutrient supplementation on structural brain development in childhood and adolescence.

## Data Availability

The raw data supporting the conclusions of this article will be made available by the author, without undue reservation.

## Footnotes

1
http://surfer.nmr.mgh.harvard.edu/
