# Supplementary material for: Randomized prenatal and postnatal nutrient supplementation shows no long-term impact on cortical gray matter in Ghanaian children
Source: Front Hum Neurosci. 2026 Jan 23;19:1672317. doi: 10.3389/fnhum.2025.1672317 (PMC12876235; doi:10.3389/fnhum.2025.1672317)
Supplement: Supplementary file 1 [file Supplementary_file_1.zip › Supplementary Material/Supplementary Figures.DOCX]

Supplementary Figure S1: Boxplots with mean and SD of cortical thickness of the right caudal anterior cingulate cortex region in the SQ-LNS group and control group.
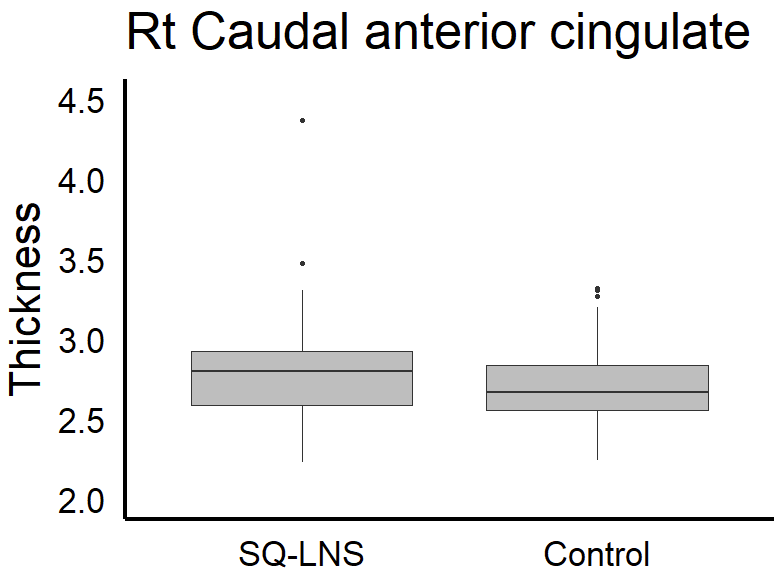
 Rt = right. SQ-LNS = Small quantity lipid-based nutrient supplementation.

Supplementary Figure S2: Boxplots with mean and SD of cortical volume of the left rostral anterior cingulate cortex region in the SQ-LNS group and control group.
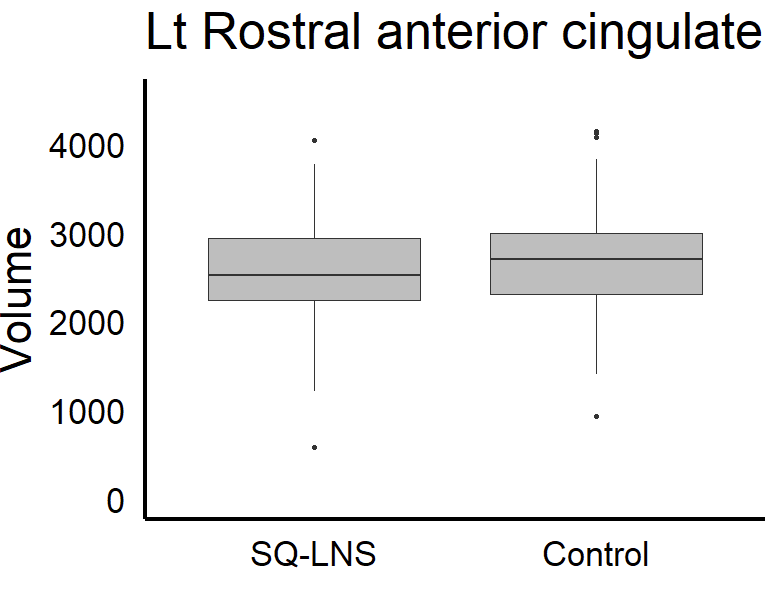
 Lt = left. SQ-LNS = Small quantity lipid-based nutrient supplementation.

Supplementary Figure S3: Boxplots with mean and SD of cortical volume of the left pallidus region in the SQ-LNS group and control group.
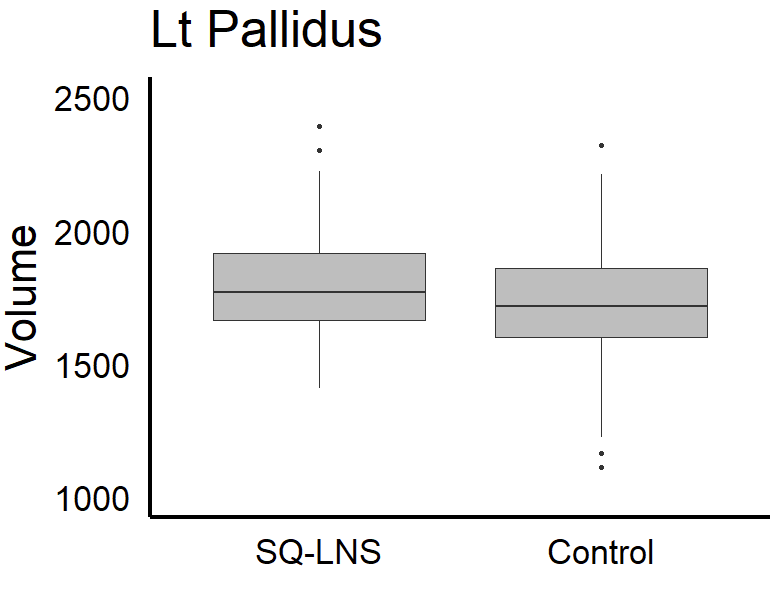
 Lt = left. SQ-LNS = Small quantity lipid-based nutrient supplementation.

Supplementary Figure S4: Boxplots with mean and SD of cortical volume of the right nucleus accumbens region in the SQ-LNS group and control group.
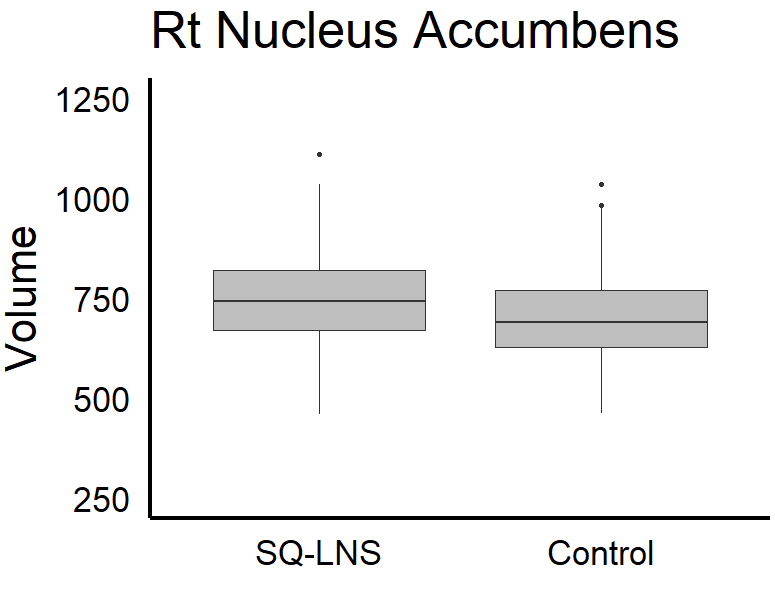
 Rt = right. SQ-LNS = Small quantity lipid-based nutrient supplementation.
