# Supplementary material for: Randomized prenatal and postnatal nutrient supplementation shows no long-term impact on cortical gray matter in Ghanaian children
Source: Front Hum Neurosci. 2026 Jan 23;19:1672317. doi: 10.3389/fnhum.2025.1672317 (PMC12876235; doi:10.3389/fnhum.2025.1672317)
Supplement: Supplementary file 1 [file Supplementary_file_1.zip › Supplementary Material/Supplementary Methods.DOCX]

**Supplementary Methods**

**Neuroimaging data, image acquisition and preprocessing**

Structural MRI data were collected using a 3T Philips Ingenia system at the Imaging department of the IMaH at Tema in Ghana, using a standard 16-channel adult-size head coil. A magnetization prepared rapid gradient echo (MPRAGE) sequence was used to create high-resolution T1-weighted images of the brain using the following parameters: turbo field echo (TFE) in 3D acquisition mode, voxel size of 1.00 x 1.00 x1.00 mm^3^, field of view (FOV) = 256 x 256 x 248, 180 sagittal slices per volume, slice thickness = 1 mm, no gap, 9-degree flip angle, repetition time (TR) = 7.3 ms, and inversion time (TI) = 944.4 ms, time to echo (TE) = 3.3 ms. The T1-weighted images were screened for quality during and after image acquisition. The raw MPRAGE images (.dcm) were converted to NIFTI format using the “dcm2nii” function from [www.nitrc.org](http://www.nitrc.org).

Debian-based Ubuntu distribution was used to run the different command tools in the BASH (Bourne-Again Shell) command language interpreter. The MPRAGE, T1-weighted data preprocessing, image reconstruction, and volumetric and cortical parcellation were carried out with the “recon-all” command from the FreeSurfer image analysis suite, version 7.2.0, (<http://surfer.nmr.mgh.harvard.edu/>). After the registration to the common template (freesurfer average-fsaverage), the data were smoothed using a 10mm 2D Gaussian smoothing kernel (1). Quality control (QC) assessments were performed visually for all 239 participants with a scan after data acquisition and preprocessing steps. The QC led to excluding neuroimaging data of participants with incomplete T1-weighted images from scans shortened due to child’s fear (n=2), and with excessive image blurring due to motion, or significant mechanical artefacts (n=2). Additionally, participants’ neuroimaging data were excluded from analysis if any neuropathology was identified in the scan by a radiologist (n=4) as shown in diagram 1.

Total intracranial volume values for each participant who passed quality control were obtained from the FreeSurfer’s automated segmentation pipeline and standardized in excel to be included in whole-brain and ROI analyses. Total gray matter volume values were obtained from the FreeSurfer output, which provided an overall measure of gray matter volume including all cortical and subcortical regions. These values were subsequently standardized before included in analyses. Additionally, cortical gray matter thickness and volume values for ROIs including left and right caudal and rostral middle frontal cortex, medial and lateral orbitofrontal cortex, caudal and rostral anterior cingulate cortex (ACC), inferior parietal cortex, and superior temporal cortex were obtained from FreeSurfer output (*N*=16 ROIs). Lastly, subcortical volume values for ROIs, including left and right amygdala, hippocampus, thalamus, caudate, pallidum, putamen, and nucleus accumbens were also obtained from the FreeSurfer output (*N*=14 ROIs). No adverse events related to MRI were reported.

1. Fischl B, Sereno MI, Tootell RBH, Dale AM. High‐resolution intersubject averaging and a coordinate system for the cortical surface. *Hum Brain Mapp*. 1999;8(4):272–84.
