# Supplementary material for: Randomized prenatal and postnatal nutrient supplementation shows no long-term impact on cortical gray matter in Ghanaian children
Source: Front Hum Neurosci. 2026 Jan 23;19:1672317. doi: 10.3389/fnhum.2025.1672317 (PMC12876235; doi:10.3389/fnhum.2025.1672317)
Supplement: Supplementary file 1 [file Supplementary_file_1.zip › Supplementary Material/Table_1.DOCX]

**Supplementary Table 1: Comparison between participants in current study and main study on baseline characteristics.**

| **Characteristics** | Total sample | **Included**  N=231 | **Excluded**  N=1089 | t-statistic | p-value |
| --- | --- | --- | --- | --- | --- |
| **Maternal** |  |  |  |  |  |
| Age at enrolment, y, mean (SD) | 26.7(5.5) | 26.84(5.5) | 26.69(5.5) | -0.4 | 0.7 |
| Educational level, n (%) |  |  |  |  | 0.05 |
| Below High school | 1084(82.1) | 200(86.6) | 884(81.2) |  |  |
| High school and above | 236(17.9) | 31(13.4) | 205(18.8) |  |  |
| Marital status n (%) |  |  |  |  |  |
| With partner | 1224(92.7) | 207(89.6) | 1017(93.4) |  | 0.05 |
| Without partner | 96(7.27) | 24(10.4) | 72(6.6) |  |  |
| Pre-pregnancy BMI, mean (SD) | 24.5(4.4) | 24.48(4.4) | 24.5(4.4) | 0.1 | 0.9 |
| Primiparous, n (%) |  |  |  |  | 0.9 |
| No previous child | 446(33.8) | 79(34.2) | 367(33.7) |  |  |
| Previous child | 874(66.2) | 152(65.9) | 722(66.3) |  |  |
| Hemoglobin level, mean (SD) |  | 111.2(12.1) |  |  |  |
|  |  |  |  |  |  |
| **Child** |  |  |  |  |  |
| Child weight at birth, kg, mean (SD) | 3.0(0.4) | 3.0(0.03) | 3.0(0.4) | -0.8 | 0.4 |
|  |  |  |  |  |  |
| **Household** |  |  |  |  |  |
| Household asset index, mean (SD) | 0.0(1.0) | 0.05(0.9) | -0.01(1.0) | -0.8 | 0.4 |
| Household food insecurity access score, mean (SD) | 2.6(4.3) | 2.17(4.0) | 2.72(4.3) | 1.8 | 0.1 |
| Household water source, n (%) |  |  |  |  | 0.4 |
| Improved | 1275(98.5) | 229(99.1) | 1046(98.3) |  |  |
| Unimproved | 20(1.5) | 2(0.9) | 18(1.7) |  |  |
| Household toilet facility, n (%) |  |  |  |  | 0.06 |
| Improved | 1262(97.5) | 220(95.7) | 1042(97.8) |  |  |
| Unimproved | 33(2.6) | 10(4.4) | 23(2.2) |  |  |
