# Supplementary material for: Randomized prenatal and postnatal nutrient supplementation shows no long-term impact on cortical gray matter in Ghanaian children
Source: Front Hum Neurosci. 2026 Jan 23;19:1672317. doi: 10.3389/fnhum.2025.1672317 (PMC12876235; doi:10.3389/fnhum.2025.1672317)
Supplement: Supplementary file 1 [file Supplementary_file_1.zip › Supplementary Material/Table_2.DOCX]

**Supplementary Table 2: Correlations among gray matter cortical thickness of regions of interest**

| **Cortical thickness** | | Caudalmiddlefrontal | | Rostralmiddlefrontal | | Lateralorbitofrontal | | Medialorbitofrontal | | Caudalanteriorcingulate | | Rostralanteriorcingulate | | Inferiorparietal | | Superiortemporal | |
| --- | --- | --- | --- | --- | --- | --- | --- | --- | --- | --- | --- | --- | --- | --- | --- | --- | --- |
|  |  | left | right | left | right | left | right | left | right | left | right | left | right | left | right | left | right |
| Caudal-middle-frontal | left | 1.0 |  |  |  |  |  |  |  |  |  |  |  |  |  |  |  |
|  | right | 0.6 | 1.0 |  |  |  |  |  |  |  |  |  |  |  |  |  |  |
| Rostral-middle-frontal | left | 0.7 | 0.6 | 1.0 |  |  |  |  |  |  |  |  |  |  |  |  |  |
|  | right | 0.5 | 0.7 | 0.7 | 1.0 |  |  |  |  |  |  |  |  |  |  |  |  |
| Lateral-orbito-frontal | left | 0.3 | 0.4 | 0.5 | 0.4 | 1.0 |  |  |  |  |  |  |  |  |  |  |  |
|  | right | 0.2 | 0.3 | 0.3 | 0.5 | 0.6 | 1.0 |  |  |  |  |  |  |  |  |  |  |
| Medial-orbito-frontal | left | 0.1 | 0.2 | 0.3 | 0.4 | 0.4 | 0.4 | 1.0 |  |  |  |  |  |  |  |  |  |
|  | right | 0.1 | 0.2 | 0.3 | 0.4 | 0.3 | 0.3 | 0.5 | 1.0 |  |  |  |  |  |  |  |  |
| Caudal-anterior-cingulate | left | 0.2 | 0.2 | 0.2 | 0.1 | 0.2 | 0.1 | 0.1 | 0.2 | 1.0 |  |  |  |  |  |  |  |
|  | right | 0.1 | 0.1 | 0.2 | 0.1 | 0.2 | 0.1 | 0.1 | 0.2 | 0.4 | 1.0 |  |  |  |  |  |  |
| Rostral-anterior-cingulate | left | 0.1 | 0.2 | 0.2 | 0.2 | 0.2 | 0.1 | 0.3 | 0.4 | 0.2 | 0.2 | 1.0 |  |  |  |  |  |
|  | right | 0.1 | 0.2 | 0.2 | 0.2 | 0.2 | 0.1 | 0.2 | 0.2 | 0.3 | 0.4 | 0.2 | 1.0 |  |  |  |  |
| Inferior-parietal | left | 0.6 | 0.4 | 0.5 | 0.4 | 0.2 | 0.1 | 0.2 | 0.2 | 0.1 | 0.1 | 0.04 | 0.2 | 1.0 |  |  |  |
|  | right | 0.4 | 0.5 | 0.4 | 0.4 | 0.3 | 0.3 | 0.2 | 0.2 | 0.1 | 0.2 | 0.1 | 0.3 | 0.6 | 1.0 |  |  |
| Superior-temporal | left | 0.3 | 0.3 | 0.3 | 0.3 | 0.4 | 0.3 | 0.3 | 0.2 | 0.2 | 0.2 | 0.2 | 0.2 | 0.2 | 0.2 | 1.0 |  |
|  | right | 0.2 | 0.4 | 0.3 | 0.4 | 0.4 | 0.4 | 0.3 | 0.2 | 0.2 | 0.2 | 0.2 | 0.2 | 0.2 | 0.3 | 0.7 | 1.0 |
