# Supplementary material for: Randomized prenatal and postnatal nutrient supplementation shows no long-term impact on cortical gray matter in Ghanaian children
Source: Front Hum Neurosci. 2026 Jan 23;19:1672317. doi: 10.3389/fnhum.2025.1672317 (PMC12876235; doi:10.3389/fnhum.2025.1672317)
Supplement: Supplementary file 1 [file Supplementary_file_1.zip › Supplementary Material/Table_4.DOCX]

**Supplementary Table 4: Correlations among gray matter volumes of subcortical regions of interest**

| **Subcortical volume** | | Amygdala | | Hippocampus | | Thalamus | | Caudate | | Pallidum | | Putamen | | Accumbens | |
| --- | --- | --- | --- | --- | --- | --- | --- | --- | --- | --- | --- | --- | --- | --- | --- |
|  |  | left | right | left | right | left | right | left | right | left | right | left | right | left | right |
| Amygdala | left | 1.0 |  |  |  |  |  |  |  |  |  |  |  |  |  |
|  | right | 0.7 | 1.0 |  |  |  |  |  |  |  |  |  |  |  |  |
| Hippocam-pus | left | 0.6 | 0.6 | 1.0 |  |  |  |  |  |  |  |  |  |  |  |
|  | right | 0.6 | 0.6 | 0.8 | 1.0 |  |  |  |  |  |  |  |  |  |  |
| Thalamus | left | 0.5 | 0.5 | 0.6 | 0.6 | 1.0 |  |  |  |  |  |  |  |  |  |
|  | right | 0.5 | 0.5 | 0.5 | 0.5 | 0.9 | 1.0 |  |  |  |  |  |  |  |  |
| Caudate | left | 0.4 | 0.4 | 0.4 | 0.4 | 0.4 | 0.4 | 1.0 |  |  |  |  |  |  |  |
|  | right | 0.4 | 0.4 | 0.4 | 0.4 | 0.4 | 0.4 | 0.9 | 1.0 |  |  |  |  |  |  |
| Pallidum | left | 0.5 | 0.5 | 0.5 | 0.5 | 0.6 | 0.6 | 0.5 | 0.5 | 1.0 |  |  |  |  |  |
|  | right | 0.5 | 0.5 | 0.5 | 0.5 | 0.7 | 0.7 | 0.5 | 0.6 | 0.9 | 1.0 |  |  |  |  |
| Putamen | left | 0.5 | 0.4 | 0.5 | 0.5 | 0.5 | 0.5 | 0.4 | 0.5 | 0.6 | 0.6 | 1.0 |  |  |  |
|  | right | 0.5 | 0.4 | 0.5 | 0.5 | 0.5 | 0.5 | 0.4 | 0.5 | 0.6 | 0.7 | 0.9 | 1.0 |  |  |
| Accumbens | left | 0.4 | 0.3 | 0.3 | 0.3 | 0.4 | 0.4 | 0.5 | 0.5 | 0.5 | 0.4 | 0.5 | 0.5 | 1.0 |  |
|  | right | 0.4 | 0.3 | 0.3 | 0.3 | 0.4 | 0.4 | 0.4 | 0.5 | 0.5 | 0.4 | 0.5 | 0.5 | 0.7 | 1.0 |
